# Mechanistic insights into DNA binding by BD1 of the TAF1 tandem bromodomain module

**DOI:** 10.1042/BCJ20253064

**Published:** 2025-09-30

**Authors:** Yogita Yadav, Phibarisha Chyne, Babu Sudhamalla

**Affiliations:** 1 Department of Biological Sciences, Indian Institute of Science Education and Research Kolkata, Mohanpur Campus, Mohanpur, West Bengal, 741246, India

**Keywords:** TAF1, tandem bromodomain, Widom 601 DNA, histones, acetyllysine

## Abstract

Transcription initiation factor TFIID subunit 1 (TAF1) is a pivotal component of the TFIID complex, critical for RNA polymerase II-mediated transcription initiation. However, the molecular basis by which TAF1 recognizes and associates with chromatin remains incompletely understood. Here, we report that the tandem bromodomain module of TAF1 engages nucleosomal DNA through a distinct positively charged surface patch on the first bromodomain (BD1). Electrostatic potential mapping and molecular docking revealed a prominent basic region on BD1 that facilitates interaction with DNA, predominantly driven by hydrogen bonds and electrostatic forces, as supported by molecular dynamics simulations. Site-directed mutagenesis identified three key positively charged residues (R1435, K1436, and R1437) within the αA helix of BD1, constituting an ‘RKR’ basic patch essential for DNA binding. Electrophoretic mobility shift assays demonstrated that the TAF1 tandem bromodomain binds DNA in a concentration-dependent manner with moderate preference for AT-rich sequences, attributed to this RKR motif. Importantly, DNA binding occurs independently of histone acetyllysine recognition by the bromodomains, as acetylated histone H4 peptides or mutations in the acetyllysine-binding pocket did not affect DNA interaction. Furthermore, nucleosome pulldown assays revealed that disruption of the BD1 RKR patch significantly reduces binding to acetylated nucleosomes, highlighting its role in facilitating chromatin engagement. Collectively, our findings establish the RKR basic patch on TAF1 BD1 as a critical determinant for DNA interaction, providing mechanistic insight into how TAF1 tandem bromodomains co-ordinate dual recognition of nucleosomal DNA and histone acetylation. These results offer a molecular basis for understanding how TAF1 may contribute to transcriptional regulation via chromatin engagement.

## Introduction

Lysine acetylation is a prevalent and crucial post-translational modification where an acetyl group is added to the ε-amino group of a lysine residue within a protein [1]. This modification is vital in controlling various cellular processes, including DNA damage, cell–cycle regulation, and chromatin remodeling [2]. Histone acetylation is commonly associated with transcriptional activation, but certain acetylation marks are also implicated in the DNA repair process [3]. Acetylated lysine residues on histones are recognized by specialized reader modules called bromodomains, which are present in various nuclear proteins and play a crucial role in regulating gene transcription by influencing chromatin structure and accessibility [4]. Depending on the protein, bromodomains can be present as single or paired tandem domains, each contributing to the recognition of acetylated lysines on histones or other proteins, thereby modulating chromatin dynamics and gene expression [4,5]. Specifically, the paired tandem bromodomains found in bromodomain and extra-terminal (BET) family proteins, such as BRD2, BRD3, BRD4, and BRDT, consist of two N-terminal bromodomains (BD1 and BD2) [4,5]. Similarly, the non-BET protein transcription initiation factor TFIID subunit 1 (TAF1) also contains tandem bromodomains, but with two C-terminal bromodomains (BD1 and BD2) [4,6]. The tandem bromodomains in BET proteins are linked by relatively long, disordered amino acid sequences, and these linkers are flexible enough to allow nucleosome scaffolding [7], whereas the tandem bromodomains in the non-BET protein TAF1 are connected by a shorter linker [4,6]. Recent studies have also revealed DNA-binding abilities in BET tandem bromodomain proteins, including BRD2 (BD1 and BD2), BRD3-BD2, BRD4 (BD1 and BD2), and BRDT-BD1 [8,9]. Additionally, BRD3-BD1 and BRDT-BD2 have been shown to weakly bind DNA, further adding complexity to their well-established role as acetyllysine readers [8,9]. Miller et al. reported that BRDT-BD1 binds double-stranded DNA (dsDNA) through a basic patch of three lysines on the αZ-helix and is proposed to adopt a bivalent mode of chromatin binding, interacting with both acetylated histones and DNA [8]. In contrast, Kalra et al. demonstrated an alternative mechanism to BRDT-BD1, in which BRD4-BD1 interacts with dsDNA through a unique nucleic acid binding pocket that partially overlaps with that of the acetyllysine-binding pocket [9]. The authors further showed that dsDNA competes with acetylated histone peptides for binding to BRD4-BD1.

This emerging theme of DNA-binding activity among bromodomain-containing proteins is not exclusive to the BET family. In fact, other bromodomain-containing proteins, such as BRG1 and BRM, central components of the BAF (mSWI/SNF) chromatin remodeling complex, also exhibit DNA-binding activity [10]. These proteins engage with DNA through a basic surface patch, suggesting that they play a role in chromatin remodeling rather than directly targeting nucleosomes [10]. Similarly, the second and fourth bromodomains of PBRM1 have been shown to interact with nucleic acids, specifically double-stranded RNA elements [11,12], indicating that these bromodomains, like their BET counterparts, may have a broader functional role that includes both acetyllysine recognition and direct interaction with nucleic acids. Given the growing evidence for DNA binding by tandem bromodomains in BET proteins and other chromatin-associated factors, we hypothesize that the tandem bromodomain of TAF1 might similarly play a role in chromatin interaction, further linking its acetyllysine-recognition function to DNA binding.

**Figure 1:**
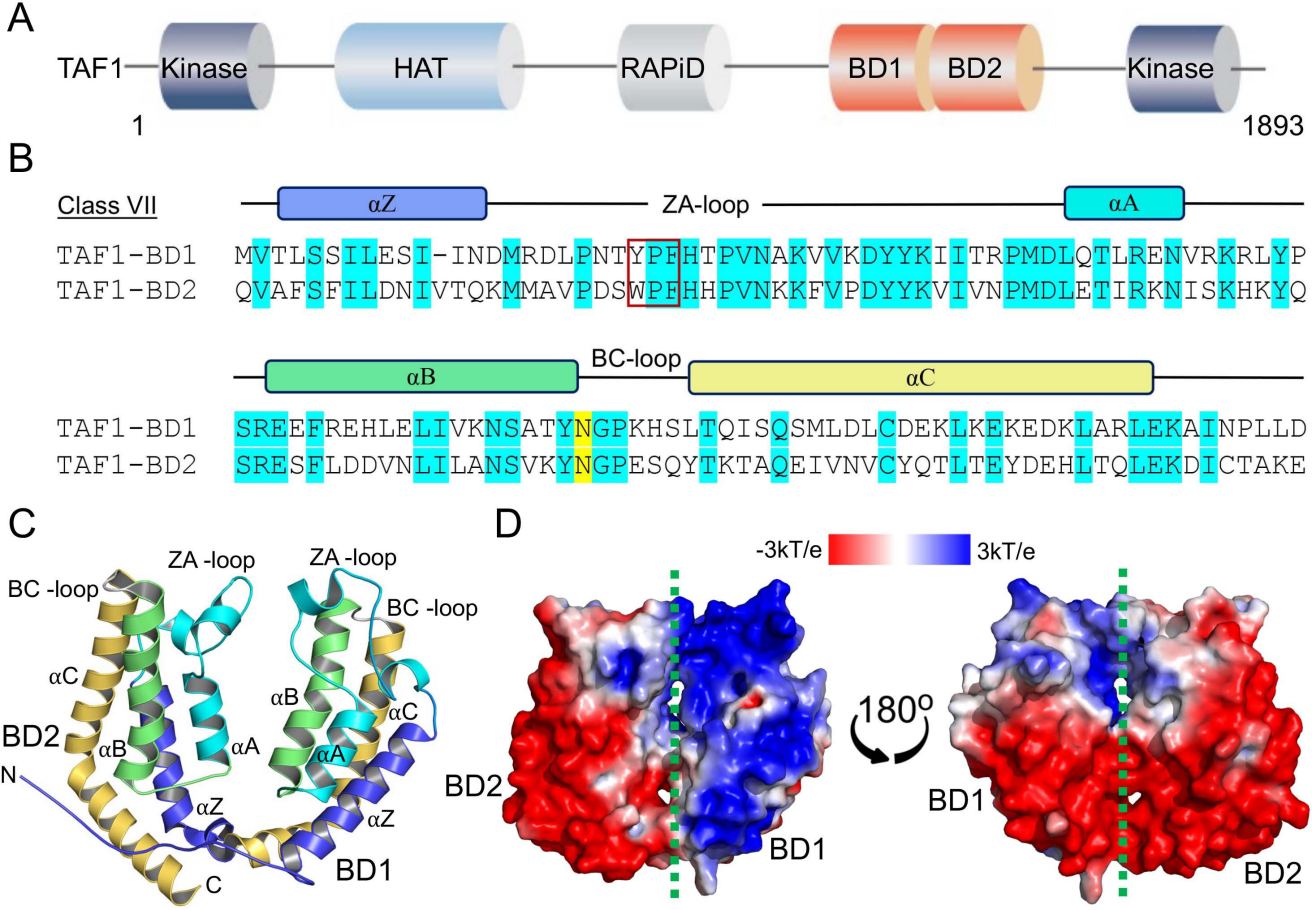
Domain organization of TAF1, sequence alignment, crystal structure, and electrostatic surface potentials of tandem bromodomain of TAF1. (**A**) TAF1 contains two kinase domains (N-terminal and C-terminal), histone acetyltransferase (HAT), RAP74 interaction domain (RAPiD), and tandem bromodomain (bromodomain 1 (BD1) and bromodomain 2 (BD2)). (**B**) The Clustal Omega alignment tool was used to show the sequence conservation between BD1 and BD2 in TAF1. The alignment indicates conserved residues important for acetyllysine binding (‘YPF/WPF’ shelf of the ZA-loop and the highly conserved asparagine ‘anchor’ residue of BC-loop), and the locations of secondary structural elements are shown and named on the top of the figure. (**C**) Cartoon representation showing the crystal structure of the TAF1 tandem bromodomain (PDB entry 1EQF). N and C termini and secondary structure elements are labeled. (**D**) The electrostatic surface potential map of the tandem bromodomain (BD1 and BD2) of TAF1 is shown, with positive and negative potentials colored in blue and red, respectively. The map illustrates the electrostatic landscape critical for ligand binding and protein–protein interactions.

TAF1 is a crucial component of the TFIID complex, required for RNA polymerase II-dependent transcription initiation [13]. TAF1 contains two kinase domains (N-terminal and C-terminal), a histone acetyltransferase domain, a RAP74 interaction domain, and tandem bromodomains (BD1 and BD2) (Figure 1A). The tandem bromodomains (BD1 and BD2) of TAF1 share an approximate sequence identity of 38% and a sequence similarity of 57%, suggesting a certain level of both functional and structural conservation (Figure 1B). Despite the relatively low overall sequence homology, both BD1 and BD2 of TAF1 share a conserved fold composed of four α-helices (αZ, αA, αB, αC), connected by variable loop regions (ZA and BC loops), which together form a largely hydrophobic acetyllysine-binding pocket [6] (Figure 1C). The crystal structure of the TAF1 tandem bromodomain determined by Jacobson et al. revealed that substrate binding specificity is influenced by the relative orientation of the two bromodomains [6]. In contrast, the structure shown by Filippakopoulos et al. demonstrated that the two bromodomains can orient independently, which may affect their ability to recognize and bind specific target sequences [4]. The tandem bromodomain of TAF1 exhibits the strongest binding to diacetylated histone H4 marks, such as H4K5acK12ac and H4K8acK16ac, and weaker binding to single acetylated H4 marks like H4K16ac [6]. Additionally, the TAF1 tandem bromodomain has been reported to bind diacetylated non-histone proteins, such as p53 (K373ac and K382ac) [14]. This interaction promotes the assembly of the TFIID complex at the *p21* gene promoter, thereby facilitating transcriptional activation. The TAF1 tandem bromodomain also binds to acute myeloid leukemia 1 (AML1)-ETO (AE) fusion transcription factor through an acetylation site at lysine 43 of AE and promotes leukemogenesis in AML cells [15]. Recent studies have shown that bromosporin-based compounds designed to inhibit the bromodomains of TAF1 exhibit significantly higher efficacy in the bivalent state compared with their monovalent counterparts [16]. However, the mechanisms by which the tandem bromodomain of TAF1 is recruited to chromatin and regulates gene transcription remain unclear.

Herein, we demonstrate that the TAF1 tandem bromodomain, specifically BD1, binds DNA via a distinct basic patch composed of three conserved, positively charged residues (R1435, K1436, and R1437) within the αA helix. This interaction shows a moderate preference for AT-rich sequences and is mediated through a surface that is distinct from the acetyllysine-binding pocket. Disruption of this basic patch impairs binding to acetylated nucleosomes, highlighting its critical role in chromatin engagement by the TAF1 tandem bromodomain.

## Results

### A positively charged region on BD1 of the TAF1 tandem bromodomain facilitates DNA binding

To investigate the structural properties of the TAF1 tandem bromodomain module, we calculated the electrostatic surface potentials using the APBS (Adaptive Poisson-Boltzmann Solver) software [17]. Our analysis revealed significant differences in electrostatic surface potentials between the two bromodomains (BD1 and BD2) of TAF1. BD1 exhibited a more positively charged surface, while BD2 showed a more negatively charged electrostatic surface potential (Figure 1D). These observations suggest that the positively charged region on BD1 could serve as a potential docking site for negatively charged DNA molecules. The stark contrast between the more positively charged BD1 and the negatively charged BD2 is intriguing and may have functional implications for DNA binding.

**Figure 2:**
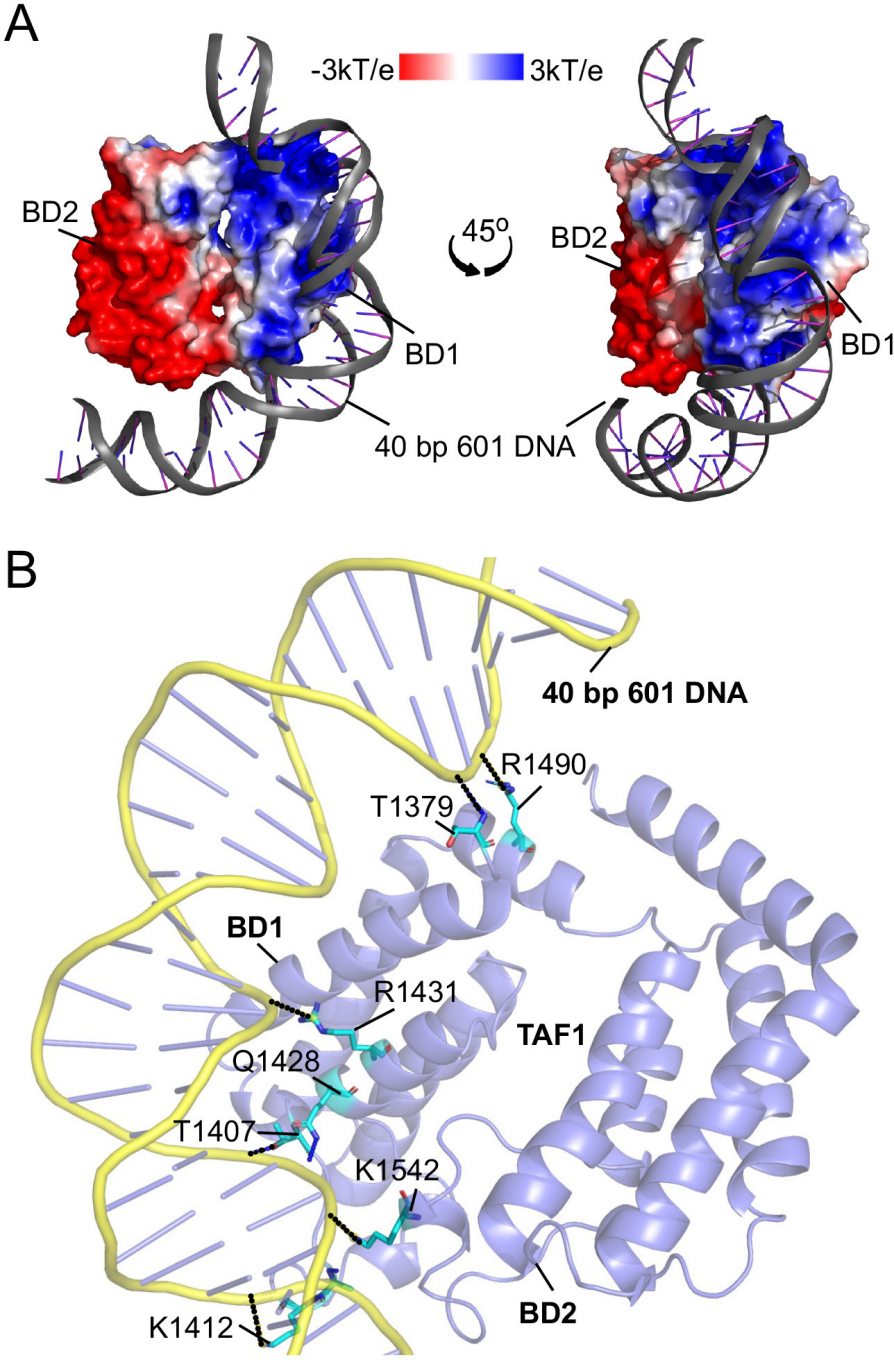
Molecular docking and molecular dynamics (MD) simulation of TAF1 tandem bromodomain binding to DNA. (**A**) The docked conformation of 40 bp 601 DNA (shown in ribbon representation) bound to the positive electrostatic potential patch of the BD1 domain of the TAF1 tandem bromodomain. The electrostatic surface potential map is overlaid, with positive regions shown in blue and negative regions in red. (**B**) MD simulation snapshot illustrating the molecular interactions between BD1 of the TAF1 tandem bromodomain and the 40 bp 601 DNA. The image highlights key contacts between BD1 and DNA, with hydrogen bonds depicted as black solid lines and salt bridges shown as black dotted lines.

To test this hypothesis, we performed molecular docking studies of the TAF1 tandem bromodomain module with 40 bp Widom 601 DNA using the HADDOCK software [18,19]. Our results showed that the most stable conformation of the 40 bp 601 DNA was docked onto the positively charged surface of BD1 (Figure 2A). This interaction was consistently observed for both the sense and antisense strands of the DNA. Additionally, protein–ligand interaction profiler (PLIP) analysis showed that the DNA primarily interacted with a centrally located, positively charged region on BD1 of the TAF1 tandem bromodomain. Positively charged residues in this region formed multiple interactions with the 601 DNA, including hydrogen bonds and salt bridges (Table 1), supporting the role of BD1’s electrostatic surface in DNA recognition.

**Table 1:** Docking summary of TAF1 tandem bromodomain with 40 bp 601 Widom DNA

Hydrogen bond		Salt bridges	
Amino acid residues	Distance (Å)	Amino acid residues	Distance (Å)
K1412	1.76	K1412	4.68
K1415	3.3	K1415	3.9
K1419	3.31	K1419	3.84
R1423	2.15	K1436	3.28
R1437	3.28	R1487	3.68
R1490	3.15	R1490	4.23

### Molecular dynamics simulation of TAF1 tandem bromodomain module–DNA complex

The best-ranked docked conformation of the TAF1 tandem bromodomain–DNA complex was subjected to 200-ns molecular dynamics (MD) simulations to further explore the DNA binding mode. Analysis of the MD trajectories using the PLIP server [20] revealed that the 601 DNA forms stable hydrogen bonds and salt bridges with several positively charged lysine and arginine residues on the surface of BD1 throughout the 200-ns simulation (Figure 2B). These findings highlight that the interaction between TAF1 and DNA is primarily driven by hydrogen bonding and electrostatic interactions. Collectively, these interactions are essential for the stable binding of the TAF1 tandem bromodomain to DNA.

### BD1 of the TAF1 tandem bromodomain module binds DNA through its αA helix

**Figure 3:**
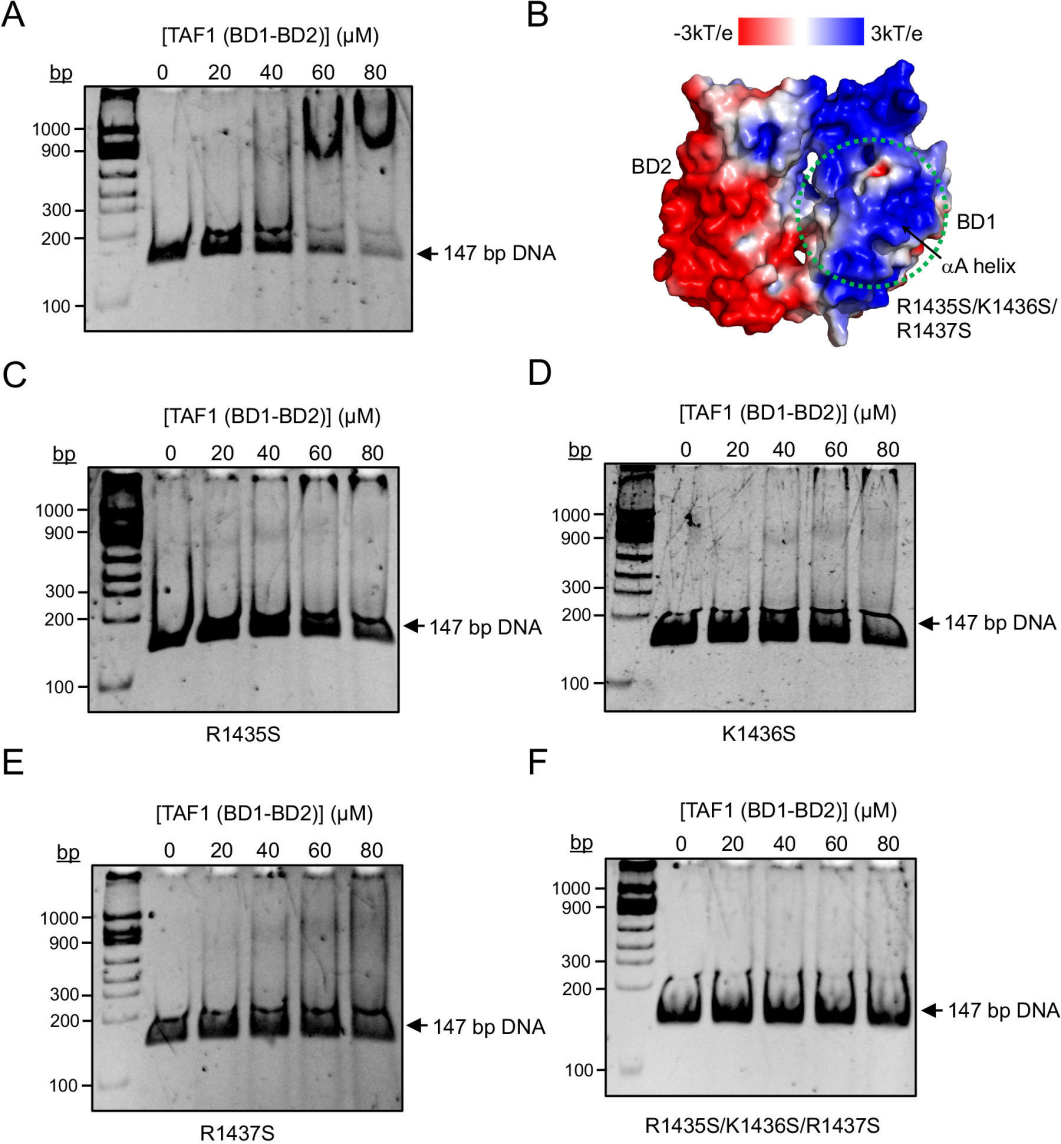
The BD1 residues of the TAF1 tandem bromodomain that are involved in DNA binding are distinct from those that recognize acetylated lysines on histones. (**A**) EMSA of TAF1 tandem bromodomain (0–80 µM) binding to 147 bp 601 DNA (35 nM). (**B**) APBS predicted a positive electrostatic patch on the BD1 of the TAF1 tandem bromodomain, centered around the α-helix (αA), which contains three key positively charged residues: R1435, K1436, and R1437. These residues were mutated to serine, both single and in combination, to probe into their role in DNA binding. (**C-F**) EMSA of BD1 mutants (R1435S, K1436S, R1437S, and R1435S/K1436S/R1437S) of TAF1 tandem bromodomain (0–80 µM) binding to 147 bp 601 DNA (35 nM).

Our *in silico* predictions suggest that the BD1 of the TAF1 tandem bromodomain interacts with dsDNA through positively charged interfaces. To determine whether the TAF1 tandem bromodomain module indeed binds dsDNA, we examined its association with 147 bp Widom 601 DNA in an electrophoretic mobility shift assay (EMSA). The 147 bp Widom 601 DNA was incubated with increasing amounts of TAF1 tandem bromodomain module, and the reaction mixtures were resolved on native polyacrylamide gels. A gradual increase in the amount of TAF1 tandem bromodomain module led to a progressive shift in the DNA band, suggesting a detectable and concentration-dependent interaction between the TAF1 tandem bromodomain module and DNA (Figure 3A and Supplementary Figure S1). These findings prompted us to investigate the specific regions within BD1 of the TAF1 tandem bromodomain responsible for DNA binding.

After a careful analysis of both the docking and MD simulation results, as well as a literature search, we identified a basic patch on the BD1 of the TAF1 tandem bromodomain module centered on the second α-helix (αA), comprising three prominent positively charged residues: R1435, K1436, and R1437 (Supplementary Figure S2). This motif is distinct but bears some resemblance to the cluster of positively charged residues (K37, K41, and K45) located in the αZ helix of BRDT-BD1, which mediate DNA binding as reported by Miller et al. [8]. To investigate the functional relevance of these BD1 residues in TAF1, we mutated each of them individually, as well as in combination, with serine to assess their contribution to DNA binding (Figure 3B and Supplementary Table S1). Interestingly, single-point mutations (R1435S, K1436S, and R1437S) each led to a marked reduction in DNA binding, with the R1435S and K1436S mutations having a more pronounced effect compared with R1437S (Figure 3C–E and Supplementary Figure S3A-C). Notably, a triple mutation (R1435S/K1436S/R1437S) completely abolished DNA binding (Figure 3F and Supplementary Figure S3D), underscoring the critical role of the αA helix and in particular these three positively charged residues in mediating the interaction between the TAF1 tandem bromodomain module and DNA.

To confirm that the impaired DNA binding observed in the single and triple mutants was not due to global misfolding or loss of protein stability, we performed circular dichroism (CD) spectroscopy on the wildtype and mutant TAF1 tandem bromodomains in the absence of DNA. The CD spectra of the R1435S, K1436S, R1437S, and the triple mutant closely resembled that of the wildtype (Supplementary Figure S4), indicating that these mutations do not significantly disrupt the secondary structure or stability of the protein.

**Figure 4:**
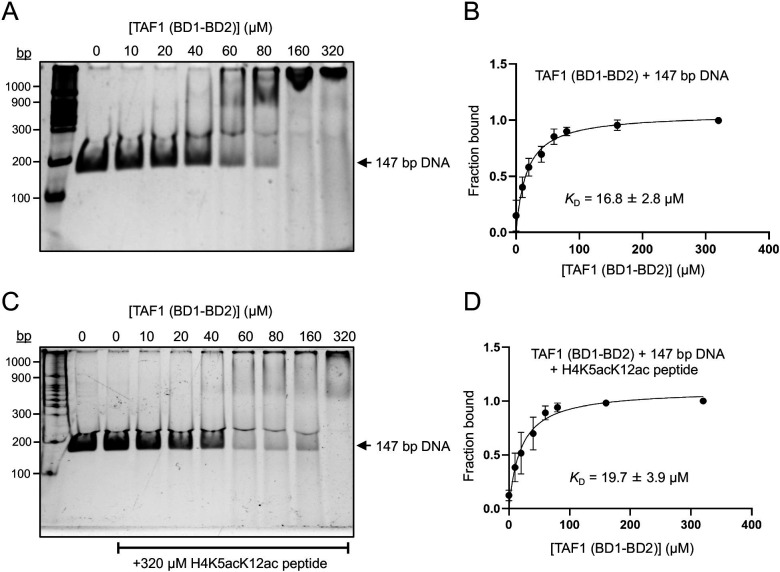
The DNA and histone tail binding interfaces of TAF1 tandem bromodomain are distinct and independent. (**A-D**) EMSA titrations of TAF1 tandem bromodomain with 147 bp 601 DNA in the absence and presence of H4K5acK12ac peptide. For binding constant measurements, 35 nM of Widom 601 DNA was incubated with varying concentrations of TAF1 tandem bromodomain (0–320 µM) in the absence and presence of 320 µM of histone H4K5acK12ac peptide for 1 h prior to native-PAGE electrophoresis at 4°C. ImageJ was used to compute the fraction bound by measuring the raw intensity density measurements of each lane. Quantification of the gel images was done in at least three independent experiments.

Considering the proximity of the TAF1 tandem bromodomain’s DNA-binding site (located on the αA helix of BD1) to its histone-binding pocket (on the ZA and BC loops), we sought to investigate whether histone tail binding could modulate its DNA-binding activity. To explore this, we performed quantitative EMSA titrations, measuring the affinity of the TAF1 tandem bromodomain for 147 bp DNA in the absence and presence of an acetylated histone H4 peptide (H4K5acK12ac). Remarkably, we observed that the binding of the acetylated H4 peptide did not significantly affect the bromodomain’s affinity for DNA, with dissociation constants (*K*_D_) of 16.8 ± 2.8 μM and 19.7 ± 3.9 μM (Figure 4A–D and Supplementary Figure S5) in the absence and presence of the histone peptide, respectively. These findings suggest that the DNA and acetyllysine-binding sites within the TAF1 tandem bromodomain function independently, without substantial interference between the two interactions.

To complement these findings, we generated the N1460F mutation in the BD1 domain of the TAF1 tandem bromodomain. To assess whether this mutation influences DNA binding, we performed EMSA using 601 DNA. Our results show that the N1460F mutation does not significantly alter DNA binding affinity. The dissociation constant for the N1460F mutant was 18.2 ± 2.3 μM (Supplementary Figure S6), which is comparable to the DNA-binding affinity of the wildtype TAF1 tandem bromodomain (*K*_D_=16.8 ± 2.8 μM). Together, these results demonstrate that the DNA-binding function of the BD1 domain within the TAF1 tandem bromodomain remains stable even upon disruption of the acetyllysine-binding pocket via the N1460F mutation. Moreover, the observation that DNA binding is unaffected by either the presence of acetylated histone H4 peptide or the N1460F mutation suggests that DNA and histone binding are distinct. This likely enables the TAF1 tandem bromodomain to engage chromatin through distinct mechanisms, contributing to its versatility in chromatin association and regulation.

### BD1 basic patch in TAF1 tandem bromodomain shows modest preference for AT-rich DNA

**Figure 5:**
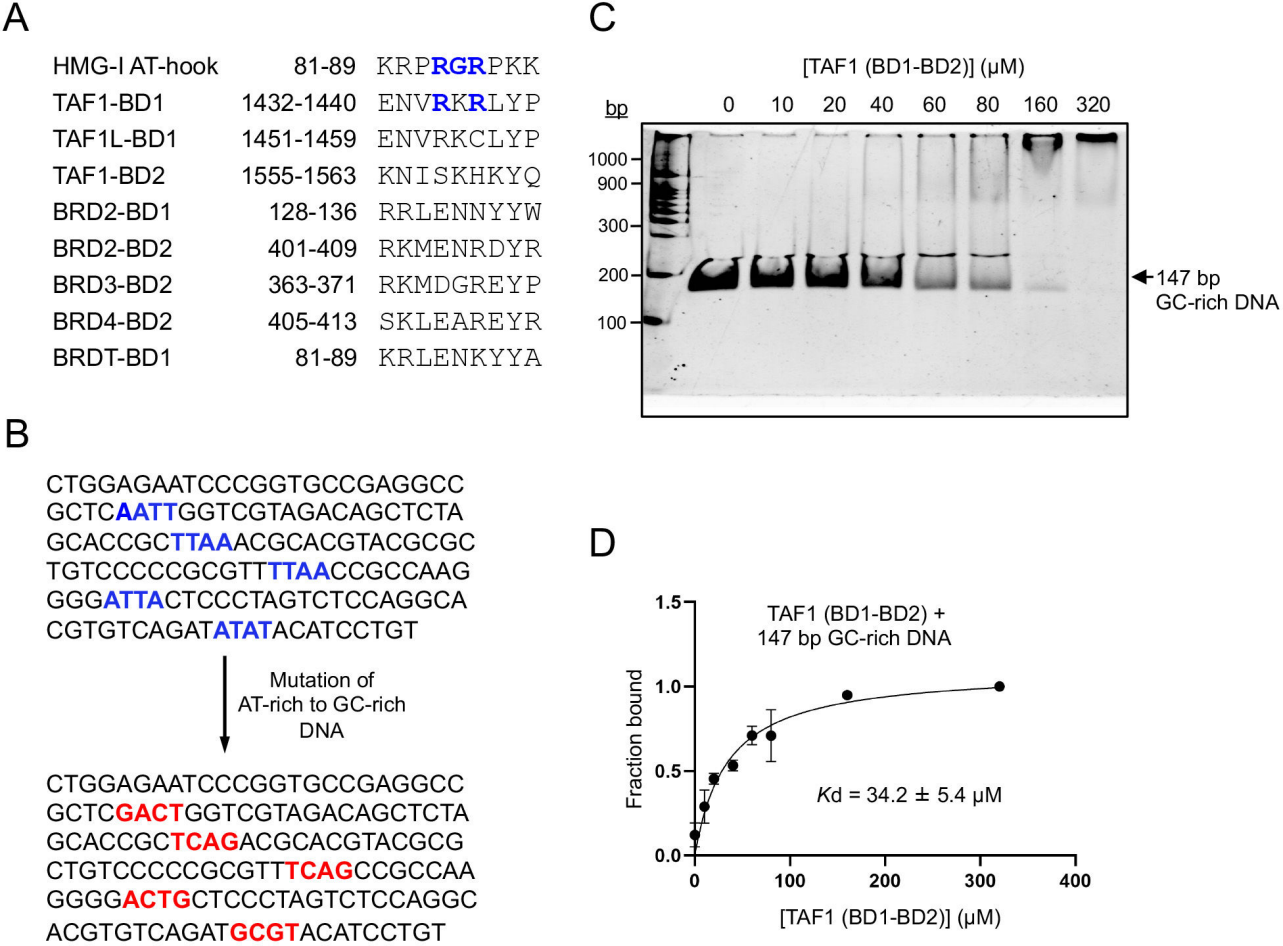
The BD1 region of TAF1 tandem bromodomains shows modest sequence-dependent DNA binding. (**A**) Sequence alignment of the HMG-1 AT-hook motif with BET (BRD2, BRD3, BRD4, and BRDT) and non-BET (TAF1 and TAF1L) bromodomain-containing proteins. The AT-hook ‘core’ region (RGR) in HMG-I and the basic patch of an AT-hook-like motif (RKR) in TAF1 are highlighted in bold and blue. (**B**) The sequence of 601 DNA with AT-tracts (4 nucleotides in length) is displayed in blue. The AT-tracts are mutated to GC nucleotides (displayed in red). (**C, D**) EMSA titrations of TAF1 tandem bromodomain with 147 bp GC-rich DNA. Binding affinities were determined by quantifying the EMSA gel images. Quantification of the gel images was done in at least three independent experiments.

Given the critical role of the RKR basic patch (R1435/K1436/R1437) in DNA binding by the BD1 of the TAF1 tandem bromodomain module, we investigated whether this feature is present in other DNA-binding proteins. Motif search analysis revealed that this basic patch (**R** K **R**) in TAF1 closely resembles the core **R** G **R** region of the AT-hook motif (P**R** G **R**P) (Figure 5A), a well-known DNA-binding motif found in high mobility group protein 1 (HMG-I), a non-histone chromatin protein with established DNA-binding function [21,22]. In canonical AT-hooks, the RGR core adopts an extended conformation and mediates direct DNA binding, while flanking proline residues contribute to structural rigidity [21]. Although the TAF1 RKR sequence lacks these flanking prolines, its similarity to the RGR core suggests a related mode of DNA engagement, albeit through a distinct structural context. To assess the conservation and uniqueness of this feature, we performed a sequence alignment across all 61 human bromodomains (Supplementary Table S2). This analysis revealed that TAF1-BD1 uniquely harbors the RKR basic patch, while TAF1L-BD1 contains a similar but non-identical RKC sequence, and GCN5L2 features RSR at the corresponding position (Supplementary Table S2). No comparable motifs were found in other bromodomains, including both BET and non-BET families. Although members of the BET family bromodomains (BRD2, BRD3, BRD4, and BRDT), which are well known for recognizing acetylated lysines on histones, have also been reported to bind DNA directly, albeit through mechanisms distinct from that of TAF1 (Figure 5A) [8,9]. These findings suggest that the RKR basic patch in TAF1-BD1 represents a unique DNA-binding adaptation within the bromodomain family, potentially indicating a distinct functional role for TAF1 in chromatin binding and gene regulation.

To assess whether the DNA-binding features observed in TAF1 are conserved in its homolog TAF1L, which shares high sequence similarity, we extended our analysis to the TAF1L tandem bromodomain. Despite the presence of a similar motif (RKC) in TAF1L-BD1, electrostatic surface potential mapping revealed a striking difference: both BD1 and BD2 of TAF1L exhibit predominantly negative surface potentials, unlike the highly positive surface of TAF1-BD1 that likely facilitates DNA interaction (Supplementary Figure S7). Furthermore, structural superimposition of TAF1 (PDB entry 1EQF) and TAF1L (AlphaFold model: AF-Q8IZX4-F1) [23,24] showed that the overall fold and positioning of the basic patch are conserved, though a cysteine replaces one of the arginines in the RKR motif (Supplementary Figure S7). This substitution may alter the DNA-binding capacity of TAF1L. While these data suggest that the unique DNA-binding potential of TAF1 may not be fully recapitulated by TAF1L, further experimental validation is required to clarify the functional implications of this sequence and electrostatic divergence.

Extending the sequence alignment analysis across all 61 human bromodomains revealed additional examples of basic residue motifs that, while not identical with the TAF1 RKR patch, share certain features suggestive of potential DNA interaction. Notably, the ‘QKK’ motif is conserved in both TRIM33A/B and TRIM66 bromodomains, and a ‘KKK’ cluster is observed in PRKCBP1. Additionally, the ‘RNH’ sequence appears in both SMARCA2A/B and SMARCA4 bromodomains (Supplementary Table S2). Although these motifs differ from TAF1’s RKR, they retain clusters of basic residues that may contribute to nucleic acid binding. To explore this possibility, we generated electrostatic surface potential maps for these bromodomains and found that these motifs localize within highly positively charged regions on the protein surfaces (Supplementary Figure S8). This electrostatic characteristic mirrors the positively charged environment surrounding the TAF1 RKR patch, which facilitates its DNA-binding ability. Taken together, these observations suggest that these bromodomains are likely candidates to investigate engagement with DNA through similar electrostatic interactions, with these basic residues possibly playing key roles in molecular recognition. However, given the structural and sequence variability, functional assays are necessary to experimentally confirm the DNA-binding capabilities of these bromodomains and to elucidate their potential roles in chromatin regulation.

To directly evaluate the DNA-binding specificity of the RKR basic patch, an AT-hook-like motif within the TAF1 tandem bromodomain, we introduced point mutations into the 147 bp Widom 601 DNA sequence to replace AT-tracts with GC-tracts (Figure 5B). We then evaluated the TAF1 tandem bromodomain’s binding to the modified 147 bp GC-rich DNA using EMSA. The resulting quantification revealed that the TAF1 tandem bromodomain interacts with the GC-rich DNA with a dissociation constant of 34.2 ± 5.4 μM (Figure 5C and D and Supplementary Figure S9), which is approximately twofold weaker compared with its binding affinity for the AT-rich DNA (*K*_D_=16.8 ± 2.8 μM) (Figure 4B). These results suggest that while the TAF1 tandem bromodomain’s basic patch can interact with both AT- and GC-rich DNA, it shows a moderate preference for AT-rich sequences, likely reflecting properties similar to AT-hook motifs, which are known to preferentially bind AT-tracts.

### BD1-mediated DNA binding enhances recognition of acetylated nucleosomes by the TAF1 tandem bromodomain

**Figure 6:**
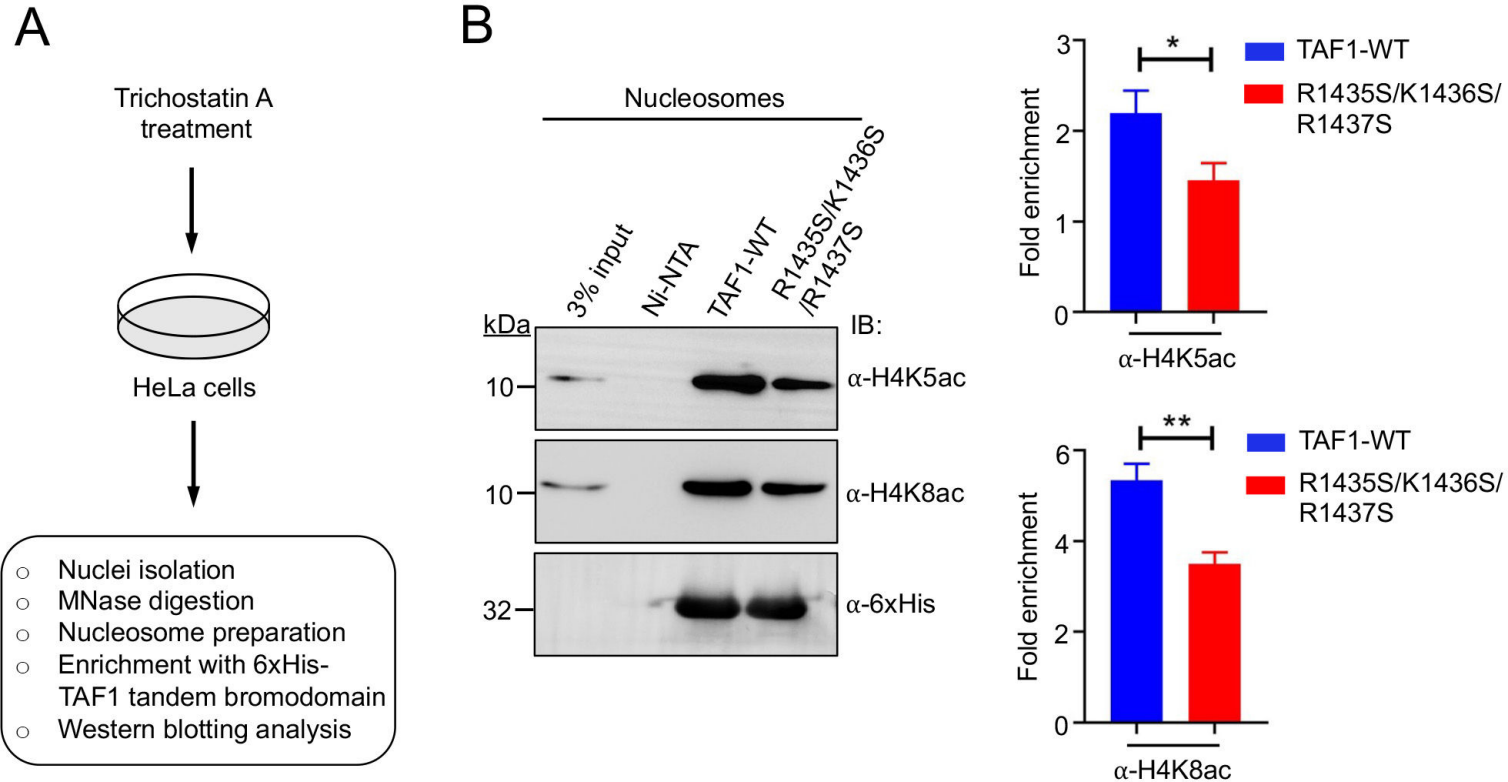
DNA binding by the BD1 of the TAF1 tandem bromodomain is crucial for acetylated nucleosome recognition. (**A**) HeLa cells were treated with the deacetylase inhibitor Trichostatin A for 24 h to induce hyperacetylation of nucleosomes. The TAF1 tandem bromodomain or its BD1 mutant was incubated with hyperacetylated mononucleosomes and enriched using Ni-NTA beads via a His-tag on the protein. The bound material was analyzed by western blotting using anti-H4K5ac and anti-H4K8ac antibodies. Immunoblotting with an anti-6xHis antibody confirmed equal loading of TAF1 proteins across the samples. (**B**) The BD1 mutant (R1435S/K1436S/R1437S) of the TAF1 tandem bromodomain exhibited a marked reduction in binding to acetylated nucleosomes compared with wildtype TAF1 tandem bromodomain. The relative band intensities were measured with ImageJ and normalized for each immunoblotting. Bars represent the mean ± standard deviation derived from three individual experiments.

To investigate whether BD1-mediated DNA binding by the TAF1 tandem bromodomain is essential for acetylated nucleosome recognition, we performed pulldown assays by incubating 6xHis-TAF1 tandem bromodomain or its BD1 mutant (R1435S/K1436S/R1437S) with acetylated mononucleosomes isolated from HeLa cells (Figure 6A and Supplementary Figure S10). The bound material was enriched using Ni-NTA beads and analyzed by western blotting. The results showed that the BD1 triple mutant (R1435S/K1436S/R1437S) exhibited a significant reduction in binding to acetylated nucleosomes, which were probed with H4K5ac and H4K8ac antibodies, with approximately a 1.5-fold decrease compared with the wildtype TAF1 tandem bromodomain (Figure 6B and Supplementary Figure S11). This reduction in binding emphasizes that the RKR basic patch of BD1 in TAF1 contributes to nucleosomal DNA interaction, facilitating efficient recognition of acetylated nucleosomes. These findings indicate that BD1-mediated DNA binding enhances the overall affinity of the TAF1 tandem bromodomain for acetylated nucleosomes, supporting a dual recognition mechanism involving both histone acetylation and DNA interaction.

## Discussion

TAF1 is the principal and largest subunit of the TFIID complex, a critical multi-protein assembly that plays a pivotal role in the initiation of transcription [13]. TAF1 contains a tandem bromodomain that specifically recognizes and binds to acetylated lysine residues on histone proteins [4]. The crystal structure of TAF1 double bromodomain, resolved at 2.1 Å resolution, reveals that the two bromodomains are aligned side by side, with a 25 Å separation between their acetyllysine-binding pockets [6]. The tandem bromodomain of TAF1 has been shown to bind multiply acetylated histone H4 peptides. Among the examined modifications, it exhibits the strongest binding affinity for the diacetylated H4K5acK12ac mark (*K*_D_=1.4 μM) and a sevenfold higher affinity for H4K8acK16ac (*K*_D_=5.3 μM) compared with the monoacetylated H4K16ac mark (*K*_D_=39 μM) [6]. This suggests that the dual bromodomain’s ability to simultaneously engage two acetylated lysines provides an added level of binding strength and specificity. However, despite these detailed peptide-level insights, there is still limited understanding of how the tandem bromodomain module of TAF1 interacts with acetylated nucleosomes in the more complex chromatin environment and how it regulates gene transcription.

In this study, we demonstrate that the tandem bromodomain module of TAF1 strongly interacts with acetylated nucleosomes and reveal a crucial role for DNA in mediating these interactions. Notably, we find that BD1 of the TAF1 tandem bromodomain interacts with DNA, and this interaction facilitates the binding of the tandem bromodomain module to acetylated nucleosomes. Through mapping the electrostatic surface potential of the tandem bromodomain of TAF1, we observe a striking contrast. BD1 displays a highly positive electrostatic surface potential, while BD2 is characterized by a more negatively charged electrostatic surface potential. Our computational docking and MD simulations further suggest that DNA binds to the positively charged region of BD1. This binding is stabilized by numerous hydrogen bonds and salt bridges with several lysine and arginine residues on the surface of BD1 within the TAF1 tandem bromodomain.

The interaction between the TAF1 tandem bromodomain and DNA was further confirmed through EMSA, providing strong evidence that the tandem bromodomain of TAF1 directly binds DNA. Notably, this interaction was not affected by the N1460F mutation in the acetyllysine-binding pocket of BD1, indicating that the two binding sites function independently. Recent studies have demonstrated an allosteric interaction between dsDNA and the first bromodomain of BRDT (BRDT-BD1) [8]. This interaction is driven by a cluster of three basic lysine residues (K37, K41, and K45) situated on the αZ-helix of the bromodomain. The presence of this basic patch facilitates the binding of dsDNA, suggesting a significant role for this region in modulating the function of BRDT. Building on these insights, we examined the TAF1 tandem bromodomain and observed a similar structural feature. In TAF1, the basic patch (R1435, K1436, and R1437) within the BD1 is centered on the second α-helix (αA). This suggests that, like BRDT-BD1, the electrostatic properties of the αA-helix in TAF1-BD1 may be pivotal for its interaction with nucleic acids. These findings suggest a conserved mechanism by which basic residues within bromodomains facilitate DNA binding, potentially influencing the broader functional roles of these proteins in cellular processes. Notably, mutation of these positively charged residues (R1435, K1436, and R1437) to serine, both individually and in combination, resulted in a complete loss of binding of the TAF1 tandem bromodomain to dsDNA. This suggests that the electrostatic interactions mediated by these residues are critical for the DNA-binding ability of TAF1-BD1. The loss of binding upon mutation further underscores the importance of the basic patch in maintaining the structural integrity and function of the bromodomain, confirming its essential role in facilitating nucleic acid interactions.

Furthermore, we observed that the binding of the acetylated histone tail to the acetyllysine-binding pocket of the TAF1 tandem bromodomain did not interfere with DNA binding by the BD1 domain. These results indicate that the DNA and acetyllysine-binding sites within the TAF1 tandem bromodomain function independently, with minimal cross-talk between the two interactions. Our findings also uncovered RKR basic patch, an AT-hook-like motif within TAF1 BD1, offering new insight into the bromodomain’s DNA-binding capabilities. AT-hooks, first identified in high-mobility group proteins such as the HMG-I(Y) protein family, which includes HMG-I, HMG-Y, and HMG-1C, are known to interact with the minor groove of AT-rich DNA sequences [21,22]. These motifs act as auxiliary DNA-binding domains, aiding in the recruitment of chromatin-associated proteins to specific regions of the genome [25]. The presence of the RKR basic patch, an AT-hook-like motif in TAF1-BD1, suggests a similar role in targeting DNA, highlighting a potential mechanism through which TAF1 contributes to chromatin engagement. Notably, the nucleosome pull-down assays with the BD1 triple mutant (R1435S/K1436S/R1437S) of the TAF1 tandem bromodomain revealed a significant reduction in binding to acetylated mononucleosomes, compared with the wildtype protein, emphasizing the functional importance of these residues in nucleosome interaction. These findings support the notion that TAF1-BD1 exhibits moderate sequence-specific DNA-binding, which facilitates the precise positioning of the tandem bromodomain on the nucleosome. This strategic arrangement enables BD1 and BD2 to interact effectively with diacetylated lysine residues (H4K5acK12ac) on histone H4 tails, thereby enhancing both the affinity and specificity of the TAF1 tandem bromodomain for nucleosomes. Overall, our results highlight a key role for BD1 in chromatin binding and suggest a mechanism by which the TAF1 tandem bromodomain recognizes nucleosomal substrates.

## Conclusion

This study highlights a distinct DNA-binding function in BD1 of the TAF1 tandem bromodomain. We show that BD1 interacts with DNA via a basic surface patch centered on the αA helix, involving three key residues, R1435, K1436, and R1437, that are essential for binding. Molecular docking and simulation studies indicate that these residues form stable electrostatic and hydrogen bond interactions with DNA. EMSA experiments further confirm this DNA-binding activity, and mutation of the RKR motif in the αA helix significantly reduces DNA binding. This interaction is independent of the acetyllysine-binding pocket, as neither the presence of acetylated histone peptides nor mutation of the acetyllysine-binding site affected BD1’s interaction with DNA. We also demonstrate that the RKR basic patch in BD1 of the TAF1 tandem bromodomain exhibits a moderate preference for AT-rich DNA sequences, although broader sequence analysis is needed to confirm this specificity. Among human bromodomains, this motif appears unique to TAF1, suggesting a specialized mode of chromatin engagement. Notably, DNA binding by BD1 enhances the interaction of the TAF1 tandem bromodomain with acetylated nucleosomes. Together, these findings support a dual interaction mechanism in which BD1 engages both DNA and acetylated histone tails through distinct, independent surfaces. This dual mode of recognition may play a critical role in TAF1’s association with chromatin and its regulation of transcription.

### Materials and methods

### Pairwise sequence alignment

The human TAF1 protein sequence (Accession: P21675) was retrieved from the NCBI database. The individual bromodomains, BD1 (residues P1381-P1497) and BD2 (residues Q1504-D1635), were aligned using the EMBOSS Needle (https://www.ebi.ac.uk/jdispatcher/psa/emboss_needle) pairwise sequence alignment tool [26].

### Calculation of electrostatic surface potentials

The crystal structure of TAF1 tandem bromodomain module (PDB entry 1EQF) was obtained from RCSB Protein Data Bank and generated the electrostatic surface potentials using PyMOL plugin APBS software [17].

### Protein-DNA docking

For protein-DNA docking studies, the crystal structure of the TAF1 tandem bromodomain module (PDB entry 1EQF) and the Widom 601 DNA (PDB entry 3LZ0) were obtained from the RCSB Protein Data Bank. The active site residues of the BD1 of the TAF1 tandem bromodomain module were chosen. Molecular docking of Widom 601 DNA with TAF1 tandem bromodomain module was performed using the HADDOCK server [18,19]. The resulting docked complexes were analyzed using PyMOL (version 2.3.4) and selected the best conformation based on the docking score for MD simulation studies.

### Molecular dynamics simulations

The top-scored docking conformation of the TAF1 tandem bromodomain module and Widom 601 DNA complex was subjected to 200 ns of MD simulations with GROMACS (version 2018.3) [27] using the CHARMM36 force field [28]. The protein–DNA complexes were solvated in a rectangular box with a distance of 20 Å between the complex and the edge of the solvated box. The solvated system was neutralized by adding sodium and chloride ions. To ensure the complex’s steric clashes or geometry, the system was energy minimized using the LINCS constraints and steepest descent algorithms (5000 steps and a force of <1000 kJ mol^−1^ nm^−1^) followed by system equilibration under *NVT* and *NPT* ensembles. The MD simulations of the complexes were carried out at 300 K and 1 atm with a 2-fs time step for 200 ns.

### Mutagenesis, expression, and purification of TAF1 tandem bromodomain

Protein expression and purification were performed using previously described methods [29]. The N-terminal 6xHis-tagged TAF1 tandem bromodomain plasmid was obtained from Addgene (a gift from Nicola Burgess-Brown, Addgene, catalog no. 39118). Subsequently, the N-terminal 6xHis-tag was deleted and inserted at the C-terminal side using the QuikChange Lightning Site-Directed Mutagenesis Kit (Agilent, catalog no. 210519). The C-terminal 6xHis-tagged TAF1 tandem bromodomain plasmid was transformed into One Shot BL21 star (DE3) *E. coli* competent cells (Invitrogen, catalog no. C601003) using pNIC28-Bsa4 kanamycin-resistant vector. A single colony was picked up and grown overnight at 37°C in 10 ml of Luria–Bertani (LB) broth in the presence of 50 μg ml^−1^ kanamycin. The culture was diluted 100-fold and allowed to grow at 37°C to an optical density (OD_600_) of 1.0. Protein expression was induced overnight at 17°C with 0.5 mM IPTG in an Innova 44 Incubator shaker (New Brunswick Scientific). Proteins were purified as follows: harvested cells were resuspended in 15 ml lysis buffer [50 mM HEPES pH 7.5, 300 mM NaCl, 5 mM β-mercaptoethanol, 5% glycerol, 20 mM imidazole, lysozyme, DNase, and 1:200 (v/v) protease inhibitor cocktail III (Calbiochem)]. The cells were lysed by pulsed sonication and centrifuged at 13000 rpm for 40 min at 4°C. According to the manufacturer’s instructions, the soluble extracts were subject to Ni-NTA agarose resin (QIAGEN, catalog no. 30210). After passing 20 volumes of washing buffer (50 mM HEPES pH 7.5, 300 mM NaCl, 5 mM β-mercaptoethanol, 5% glycerol, and 20 mM imidazole), proteins were eluted with a buffer containing 50 mM HEPES pH 7.5, 300 mM NaCl, 5 mM β-mercaptoethanol, 5% glycerol, and 300 mM imidazole. Proteins were further purified by gel filtration chromatography (Superdex-75) using AKTA pure FPLC system (GE Healthcare) with a buffer containing 50 mM HEPES pH 7.5, 200 mM NaCl, and 5% glycerol. Purified proteins were concentrated using Amicon Ultra-10k centrifugal filter device (Merck Millipore Ltd.), and the concentration was determined using Bradford assay kit (Bio-Rad Laboratories) with BSA as a standard. The proteins were aliquoted and stored at −80°C before use.

Point mutations including R1435S, K1436S, R1437S, the triple mutant R1435S/K1436S/R1437S, and N1460F (Supplementary Table S1) located in the acetyllysine-binding pocket were introduced into the BD1 region of the pNIC28-Bsa4 TAF1 tandem bromodomain construct using the QuikChange Lightning Site-Directed Mutagenesis Kit, following the manufacturer’s protocol. All mutant plasmids were verified by DNA sequencing. Mutant proteins were expressed and purified using the same procedures established for the wildtype TAF1 tandem bromodomain.

### 147 bp Widom 601 DNA amplification and purification

A plasmid containing 147 bp with a centered Widom 601 sequence (pGEM-3z/601) was obtained from Addgene (a gift from Jonathan Widom, Addgene, catalog no. 26656) [30]. The 147-bp nucleosome positioning sequence was amplified by performing PCR using the following primers: For 5′-CTGGAGAATCCCGGTGCCG-3′ and Rev 5′-ACAGGATGTATATATCTGACACG-3′ used by the Widom lab. The Phusion High-Fidelity DNA Polymerase (NEB, catalog no. M0530S) was used for amplifying 147 bp Widom 601 DNA following manufacturer’s protocol. The PCR product was separated on 1.5% agarose gel and extracted with the QIAquick Gel Extraction Kit (QIAGEN, catalog no. 28704). The purified DNA was aliquoted and stored at −20°C before use.

### Mutagenesis of 147 bp Widom 601 DNA

Point mutations were introduced into the pGEM-3z/601 DNA construct to generate GC-rich dinucleotides in 147 bp Widom 601 DNA using the QuikChange Lightning Site-Directed Mutagenesis Kit. The resulting mutant plasmids were confirmed by DNA sequencing. All the mutants were PCR-amplified and purified as described above.

### Electrophoretic mobility shift assay

For the binding reaction, a master mix containing DNA and reaction buffer (50 mM HEPES pH 7.5, and 100 mM NaCl) was prepared and mixed with dilutions of TAF1 tandem bromodomain constructs to the final concentrations indicated with a total reaction volume of 20 μl. The final concentration of Widom 601 DNA used in each lane is 35 nM against varying concentrations of wildtype TAF1 tandem bromodomain module or its mutants (0–80 µM) along with a 100-bp DNA ladder (Bio Bharati Life Science, catalog no. BB-D50). Reaction mixtures were incubated for 1 h at 4°C (2.5 µl loading dye was added to each sample) and loaded onto a 6% native polyacrylamide gel. Electrophoresis was performed in 0.5× TBE buffer at 100 V on ice. Gels were stained with ethidium bromide (10 mg/ml) for 20 min and imaged using the Syngene G:BOX Chemi-XRQ imaging system.

For binding constant measurements, 35 nM of Widom 601 DNA was incubated with varying concentrations (0–320 µM) of either the TAF1 tandem bromodomain module or its BD1 mutant (N1460F) for 1 h at 4°C. For the wildtype protein, parallel reactions were performed in the absence or presence of 320 µM histone H4 (1–15) K5acK12ac peptide (SGRGKacGGKGLGKacGGA). The N1460F mutant was tested under the same protein and DNA concentrations, but without peptide. The samples were loaded onto a 6% native polyacrylamide gel, and electrophoresis was performed in 0.5× TBE buffer at 100 V on ice. Gels were stained with SYBR Gold (Invitrogen, catalog no. S11494) and imaged as described above. ImageJ was used to compute the fraction bound by measuring the raw intensity density measurements of each lane. Quantification of the gel images was done for at least three independent experiments. The binding constant (*K*_d_) values were determined using a non-linear regression equation:


$$Y=\frac{Bmax*X}{\left(Kd+X\right)}$$

where *X* is the concentration of protein, *Y* is the fraction bound calculated from our raw intensity density measurements, and *B_max_* is the maximum binding in the same units as *Y* and *K*_d_ is the binding constant. The *K*_d_ values reported are averaged out of three experiments, and the error is calculated as the standard deviation between the three replicates.

### Circular dichroism spectroscopy

The CD) experiment was performed by following the method described previously [31]. Briefly, spectra were recorded on a JASCO J-1500 CD spectrometer at 20°C using a quartz cell with a path length of 10 mm. Two scans were accumulated at a scan speed of 100 nm min^−1^, with data being collected every nanometer from 195 nm to 260 nm. Wildtype TAF1 tandem bromodomain and its mutant proteins were diluted to 2.5 µM CD buffer containing 150 mM NaCl and 50 mM NaH_2_PO_4_ (pH 7.0), and the spectra were recorded. The ellipticity data were converted to molar ellipticity using the following equation:


$$\left[\Theta ]=m^{o}M/(10LC\right)$$

where [Θ] is the molar ellipticity, *m^o^* is the ellipticity of the measured sample, *M* is the average molecular weight (g/mol), *L* is the path length of the cell (cm), and *C* is the concentration (g/l).

### Mammalian cell culture

HeLa cells were grown in DMEM supplemented with 10% fetal bovine serum and antibiotics (penicillin/streptomycin cocktail) in a humidified atmosphere containing 5% CO_2_. Cells at ~90% confluence were treated with 2 μM histone deacetylase inhibitor, trichostatin A (Sigma, catalog no. T8552) dissolved in dimethyl sulfoxide to generate hyperacetylated histones. Twenty hours post-treatment, medium was removed and the cells were rinsed with cold phosphate-buffered saline (PBS). The harvested cells were rewashed with cold PBS and frozen as a dry pellet at −80°C.

### Isolation of mononucleosomes from HeLa cells

Mononucleosomes were isolated from HeLa cells by following the protocol as previously described [32,33]. Briefly, the cells were harvested post-treatment with 2 μM histone deacetylase inhibitor (TSA) for 20 h and washed twice in PBS. The cells were resuspended in buffer A [10 mM HEPES (pH 7.9), 10 mM KCl, 1.5 mM MgCl_2_, 340 mM sucrose, 10% (v/v) glycerol, protease inhibitor cocktail (Pierce), 1 μg/ml TSA, and 5 mM 2-mercaptoethanol] and an equivalent volume of buffer A supplemented with 0.1% (v/v) Triton X-100 detergent and incubated on ice for 10 min. The nuclei were pelleted by centrifugation (1300 g for 5 min at 4°C) and washed twice with ice-cold buffer A, followed by centrifugation (1300 g for 5 min at 4°C), and the supernatant was carefully removed. The nuclei were resuspended in a buffer containing 50 mM Tris-HCl (pH 8.0) and 5 mM CaCl_2_. The suspension of the nuclei was preincubated at 37°C for 5 min and then digested with 500 U micrococcal nuclease (Invitrogen, catalog no. 88216) was carried out for 30 min as per the manufacturer’s protocol. To facilitate the release of digested nucleosomes, EGTA (pH 8.0) was added to final concentrations of 20 mM. The suspension of the nuclei was preincubated at 37°C for 5 min and then digested with 500 U micrococcal nuclease (Invitrogen, catalog no. 88216) by following the manufacturer’s protocol. To facilitate the release of digested nucleosomes, NaCl was added to final concentrations of 200 mM, and reaction mixtures were spun down at 1300 g for 5 min at 4°C. The supernatant-containing soluble nucleosomes were collected and stored at −80°C until use.

### Ni-NTA pull-down assays and western blotting

Pulldown experiments were performed by following the method previously described [31,34]. First, 20 μg of mononucleosomes was mixed with 50 μM recombinant wildtype 6xHis-tagged TAF1 tandem bromodomain or its mutant and incubated at 4°C on a nutator for 60 min. Samples were transferred to a fresh tube containing 80 μl of Ni-NTA agarose (QIAGEN) and incubated for 60 min at 4°C on a nutator. The beads were then washed five times with 1 ml of wash buffer containing 50 mM HEPES (pH 7.5), 500 mM KCl, 2 mM EDTA, and 0.1% Tween-20. Bound nucleosomes were eluted by incubating the beads in 20 μL of elution buffer [50 mM HEPES (pH 7.5) and 500 mM imidazole] at 4°C on a nutator. Equal volumes of eluted samples were separated on a 12% sodium dodecyl sulfate-polyacrylamide gel electrophoresis gel and transferred onto a 0.45 μm PVDF membrane at a constant voltage of 80 V for 1 h at 4°C. The membrane was rinsed in TBST buffer [50 mM Tris (pH 7.4), 200 mM NaCl, and 0.1% Tween 20] and blocked for 1 h at room temperature in 5% milk buffer prepared in TBST. Immunoblotting was performed overnight at 4°C with the primary antibodies, H4K5ac (Invitrogen, catalog no. MA532009), and H4K8ac (Invitrogen, catalog no. MA533386). The membranes were washed with TBST buffer thrice at room temperature for 5 min each. The blots were then incubated with the HRP-conjugated secondary antibody goat anti-rabbit IgG (Invitrogen, catalog no. 31466) with 5% nonfat dry milk, diluted 1:20,000 in TBST. The membranes were rewashed with TBST buffer thrice at room temperature for 5 min each. Protein bands were visualized by chemiluminescence using SuperSignal West Femto substrate (Invitrogen, catalog no. 34094) following the manufacturer’s protocol.

## Supplementary material

Online supplementary material 1

Online supplementary table 1

## Data Availability

The authors declare that the main data supporting the findings of this study are available within the article and its Supplementary materials.

## Abbreviations

AML: acute myeloid leukemia
APBS: Adaptive Poisson-Boltzmann Solver
BD: bromodomain
BET: bromodomain and extra-terminal
CD: circular dichroism
EMSA: electrophoretic mobility shift assay
HMG-I: high mobility group protein 1
MD: molecular dynamics
MNase: micrococcal nuclease
PBS: phosphate-buffered saline
PLIP: protein-ligand interaction profiler
TAF1: transcription initiation factor TFIID subunit 1
TSA: trichostatin A
