# Supplementary material for: Mechanistic insights into DNA binding by BD1 of the TAF1 tandem bromodomain module
Source: Biochem J. 2025 Sep 30;482(19):1415–29. doi: 10.1042/BCJ20253064 (PMC12599243; doi:10.1042/BCJ20253064)

**Supporting Information**

**Mechanistic insights into DNA binding by BD1 of the TAF1 tandem bromodomain module**

Yogita Yadav^1,a^, Phibarisha Chyne^1,a^, and Babu Sudhamalla^1,^*

^1^Department of Biological Sciences, Indian Institute of Science Education and Research Kolkata, Mohanpur Campus, Mohanpur, 741246 Nadia, West Bengal, India.

^a^These authors contributed equally

*Corresponding author: Babu Sudhamalla

**Email:** s.babu@iiserkol.ac.in

**Table of Contents**

1. Supplementary Figures S2-S10
2. Supplementary Tables S11


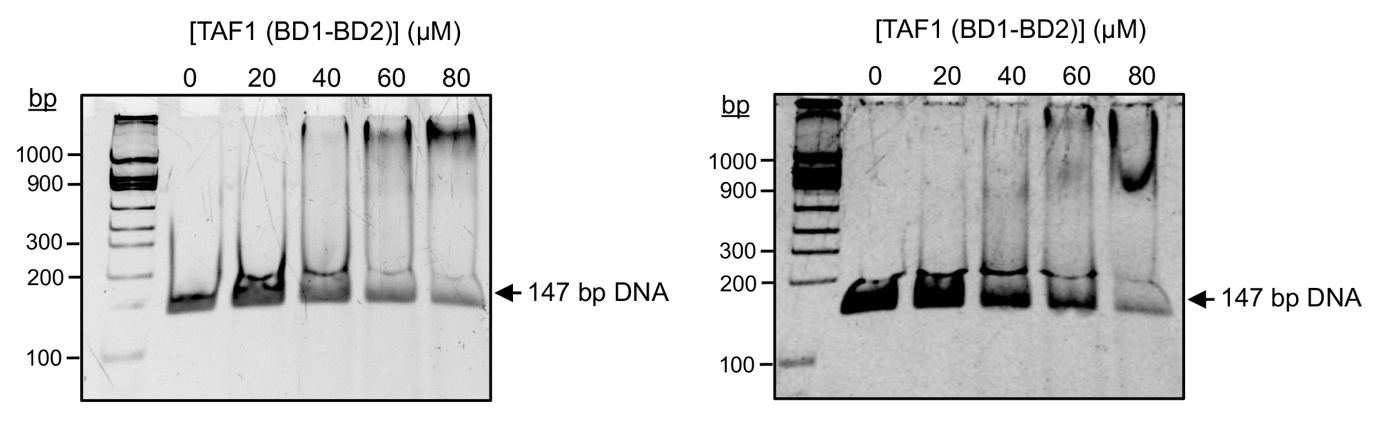


**Figure S1.** Interaction of TAF1 tandem bromodomain with DNA. EMSA of TAF1 tandem bromodomain (0-80 µM) binding to 147 bp 601 DNA (35 nM). Data from biological replicates are shown.


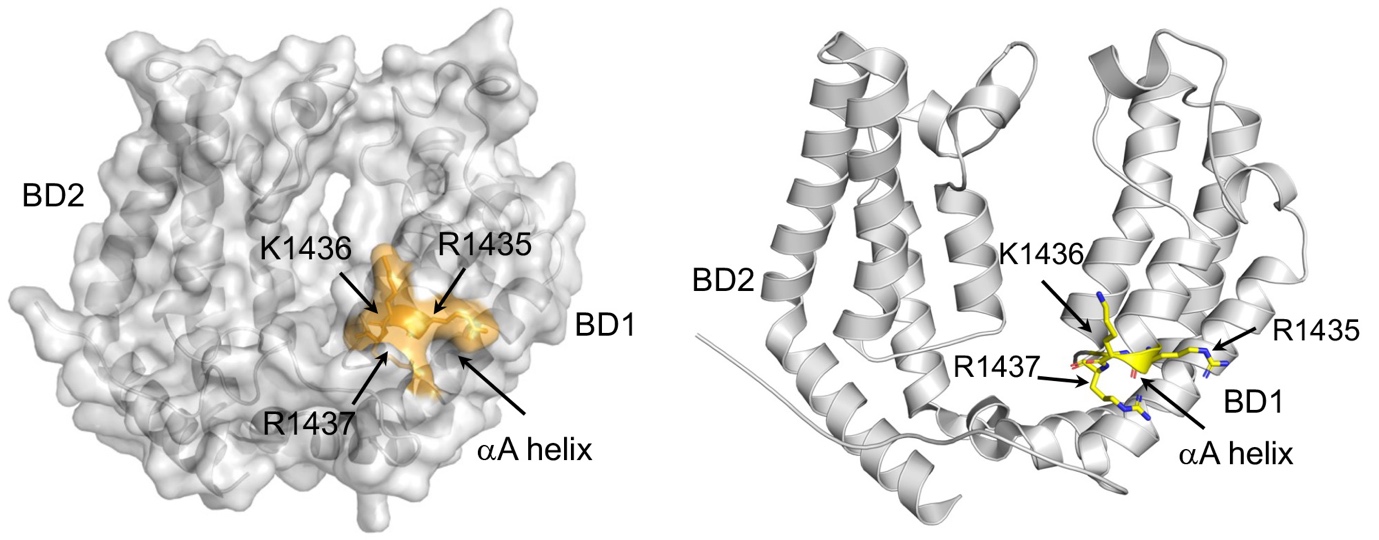


**Figure S2.** APBS (Adaptive Poisson-Boltzmann Solver) predicted a positive electrostatic patch on the BD1 of the TAF1 tandem bromodomain, centered around the α-helix (αA), which contains three key positively charged residues: R1435, K1436, and R1437. This motif is distinct but bears some resemblance to the cluster of positively charged residues (K37, K41, and K45) located in the αZ helix of BRDT-BD1 (PDB code – 2RFJ), which mediate DNA binding as reported by (Miller et al. Nature Communications. 2016, 7, 13855).


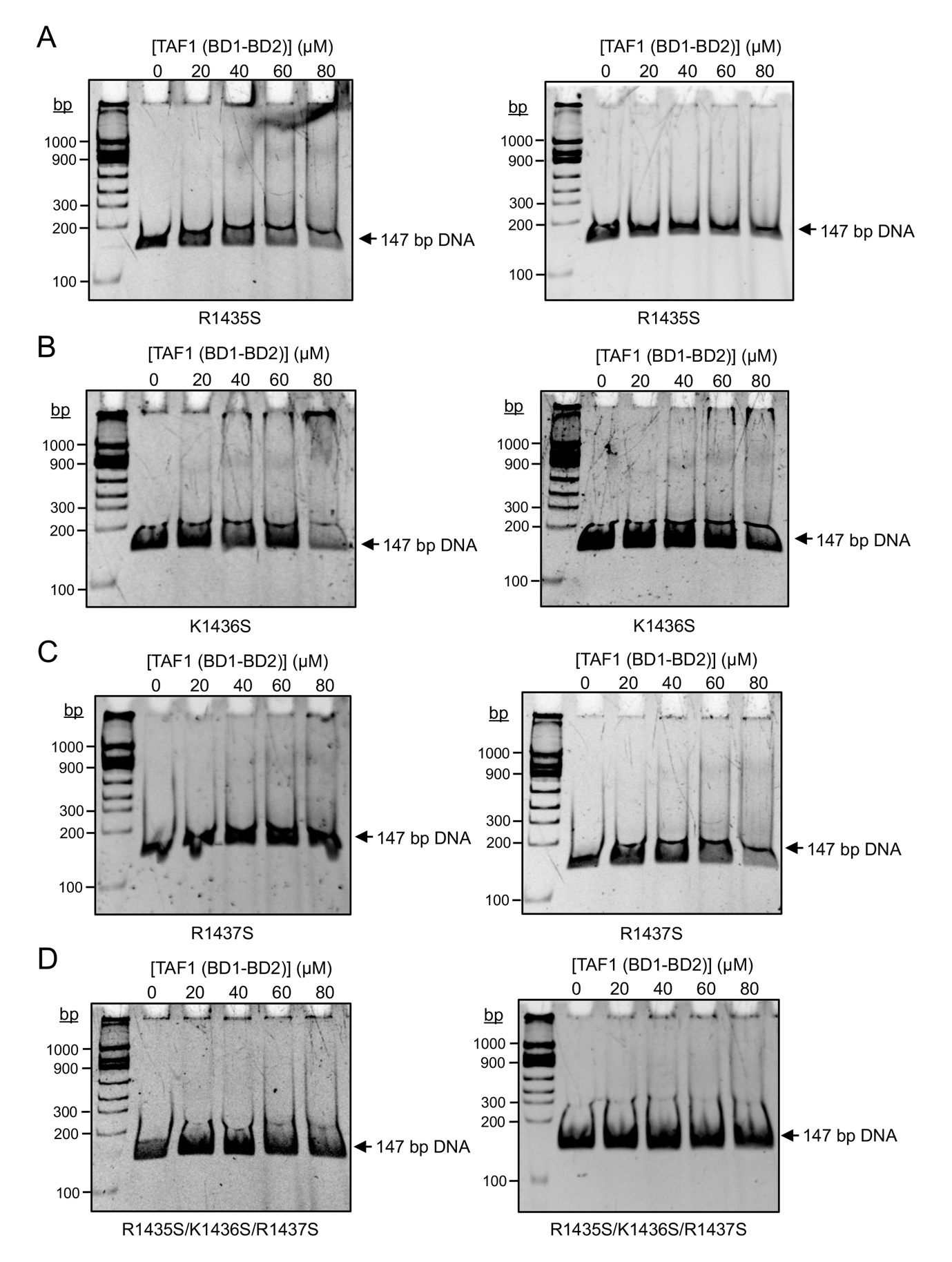


**Figure S3.** Specific positively charged residues within the αA-helix region of TAF1-BD1 are essential for DNA binding. (A-D) EMSA of BD1 mutants (R1435S, K1436S, R1437S and R1435S/K1436S/R1437S) of TAF1 tandem bromodomain (0-80 µM) binding to 147 bp 601 DNA (35 nM). Data from biological replicates are shown.

**
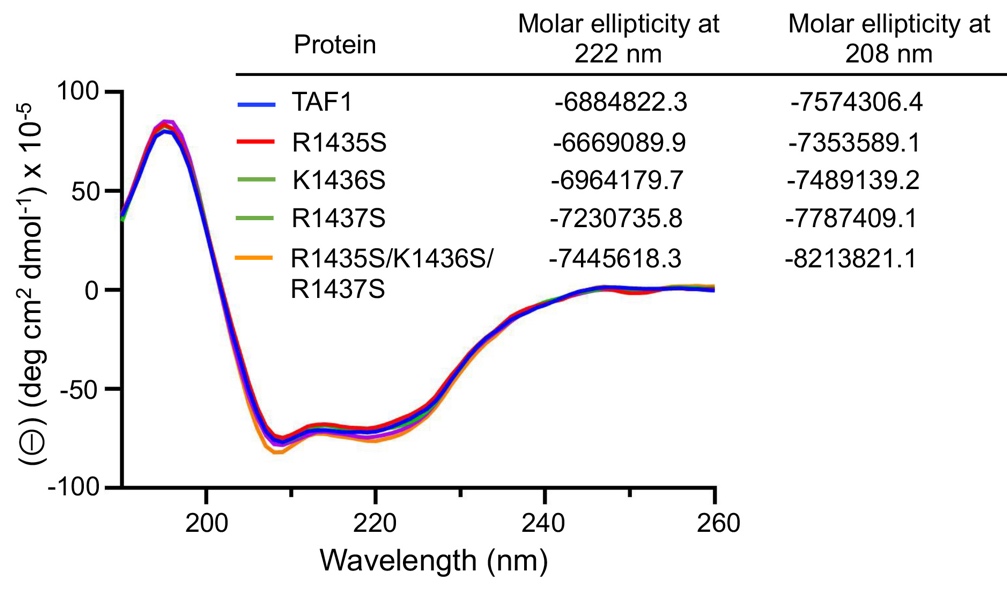
**

**Figure S4.** Circular dichroism (CD) analysis of wild-type and mutant TAF1 tandem bromodomains (R1435S, K1436S, R1437S, and the triple mutant R1435S/K1436S/R1437S) indicates that these mutations do not significantly alter the protein’s secondary structure.


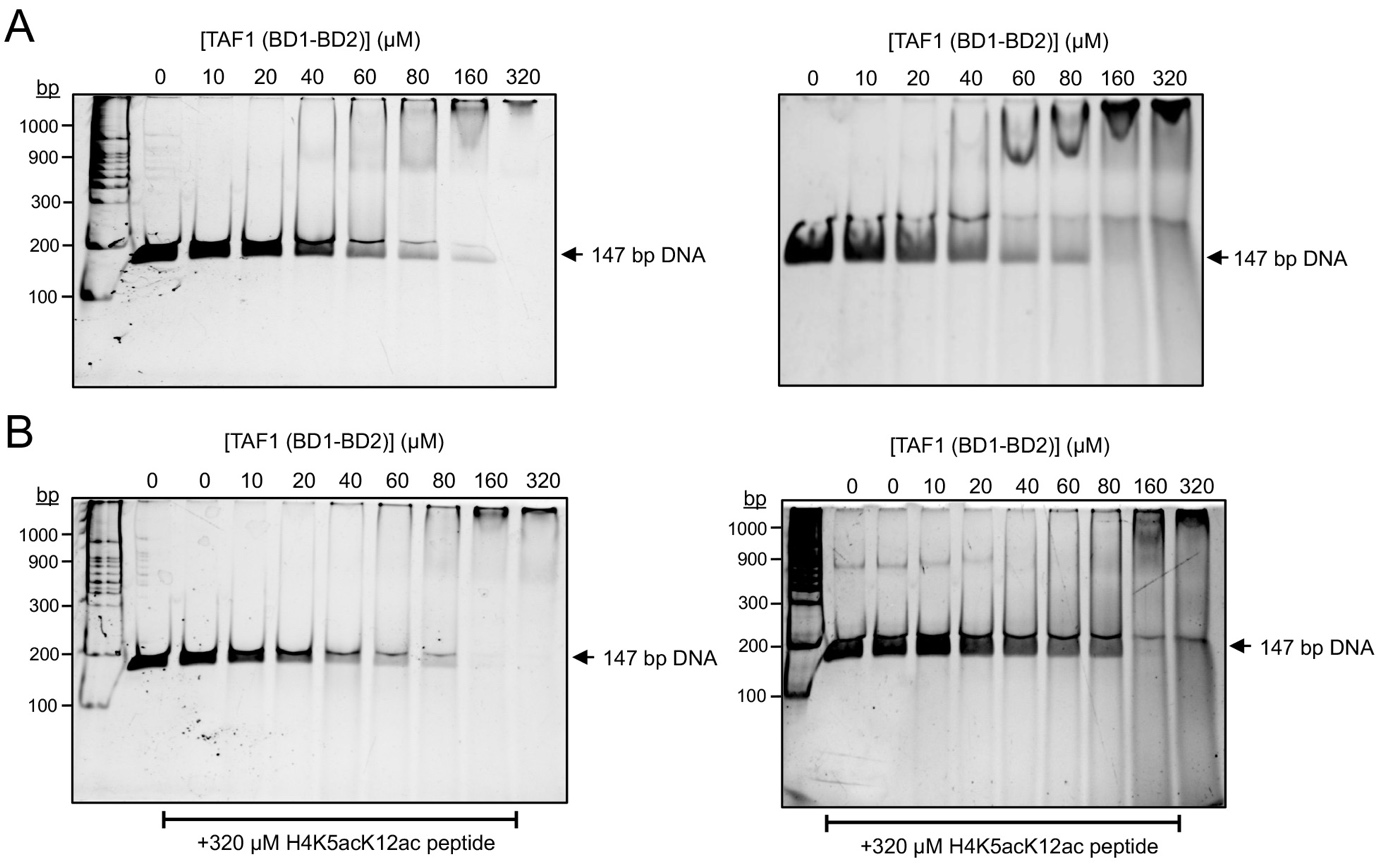


**Figure S5.** The DNA and histone tail binding interfaces of TAF1 tandem bromodomain are distinct and independent. (A, B) EMSA titrations of TAF1 tandem bromodomain with 147 bp 601 DNA in the absence and presence of H4(1-15)K5acK12ac peptide (SGRGKacGGKGLGKacGGA). For binding constant measurements, 35 nM of Widom 601 DNA was incubated with varying concentration of TAF1 tandem bromodomain (0 to 320 µM) in presence or absence of 320 µM of histone H4K5acK12ac peptide for 1 h prior to native-PAGE electrophoresis at 4 °C. Data from biological replicates are shown.

**
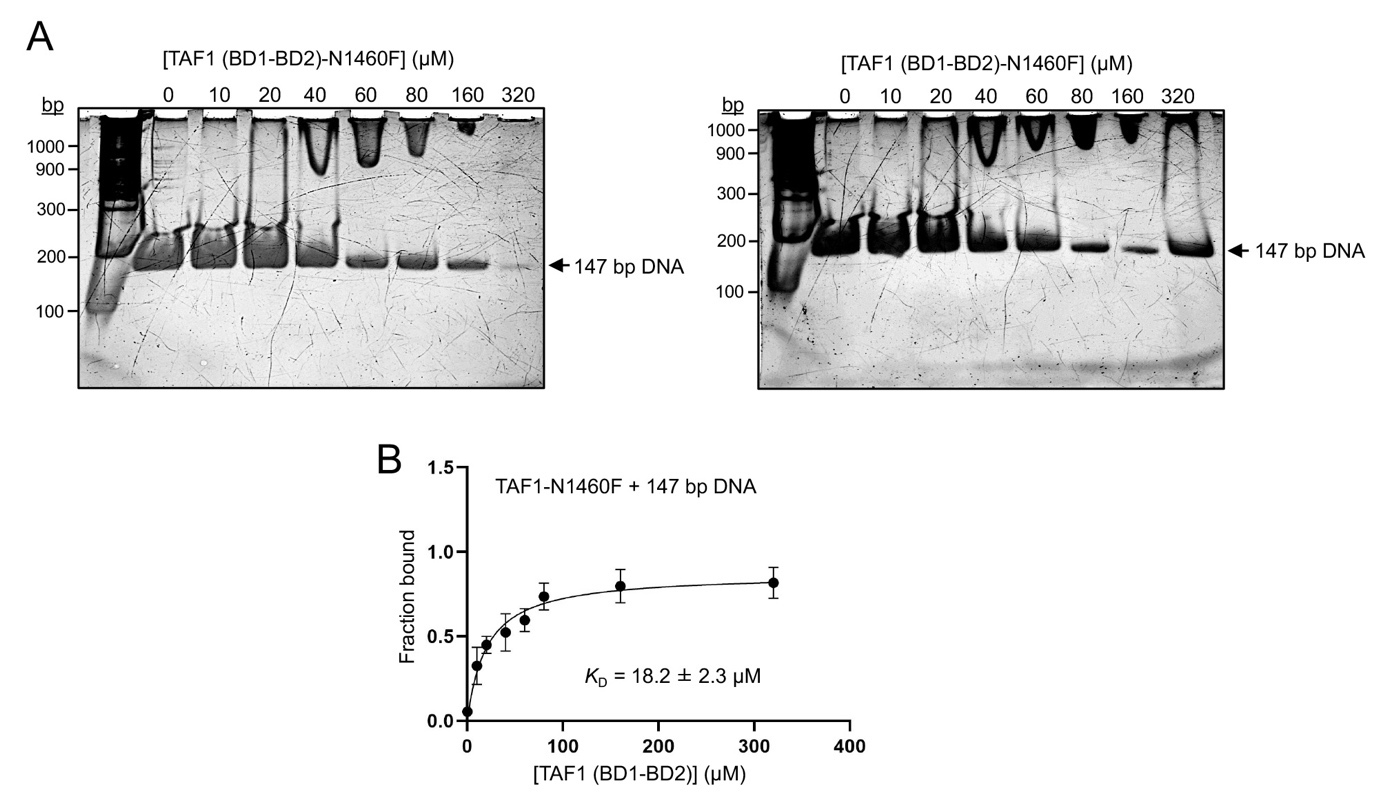
**

**Figure S6.** The BD1 N1460F mutation does not affect DNA binding by the TAF1 tandem bromodomain. (A) Electrophoretic mobility shift assay (EMSA) of the TAF1 tandem bromodomain BD1 N1460F (PDB code: 1EQF) mutant with 147 bp Widom 601 DNA. Increasing concentrations of the mutant protein (0-320 µM) were incubated with 35 nM DNA for 1 hour at 4 °C, followed by native-PAGE. (B) Quantification of DNA binding from EMSA gels. Band intensities were used to generate binding curves. Data represent biological replicates.

**
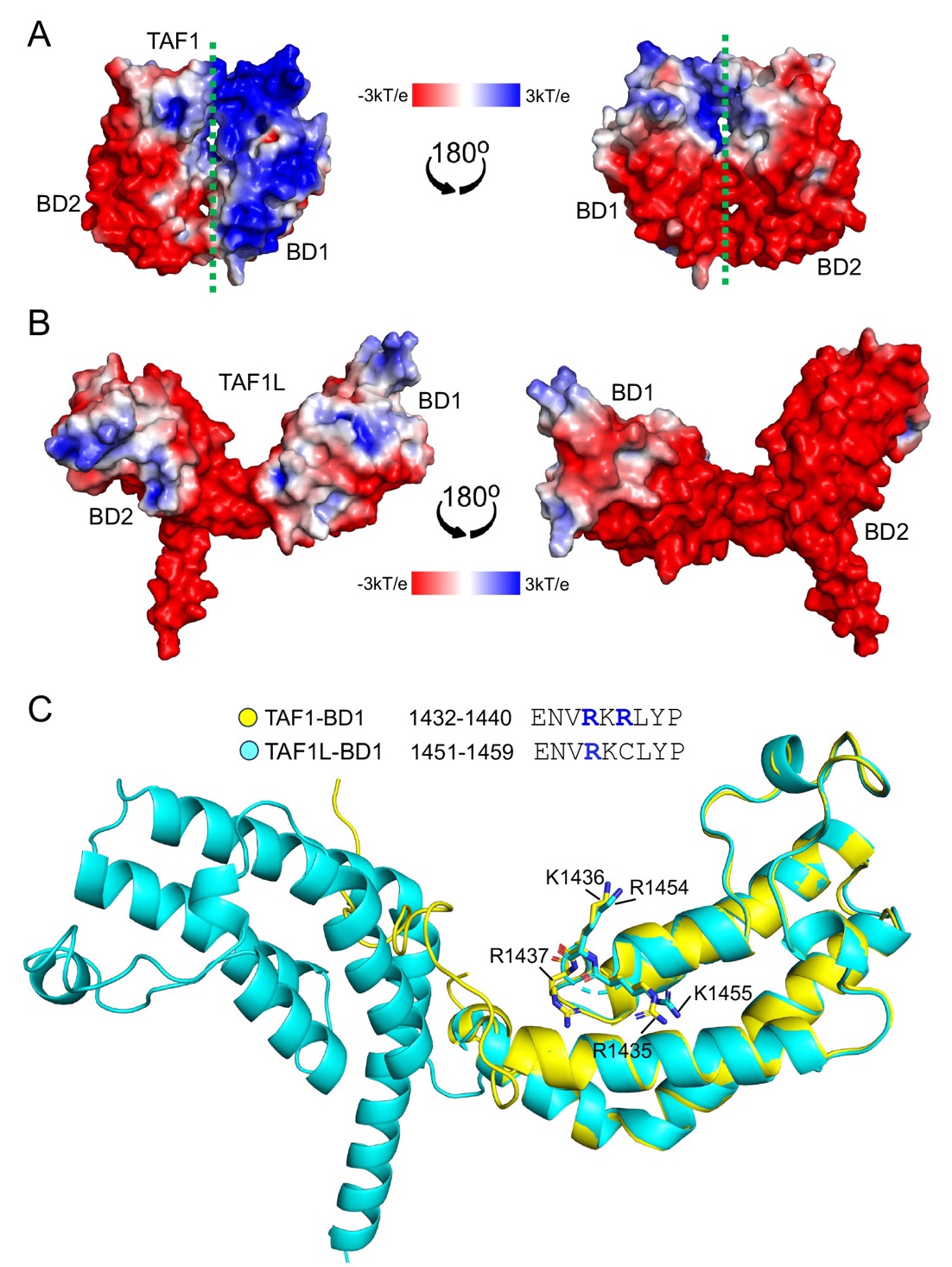
**

**Figure S7.** Comparative electrostatic surface potentials and structural alignment of TAF1 and TAF1L tandem bromodomains. **(A, B)** Electrostatic surface potential maps of the TAF1 (A) and TAF1L (B) tandem bromodomains, illustrating distinct charge distributions. Both BD1 and BD2 of TAF1L exhibit predominantly negative surface potentials, whereas BD1 of TAF1 tandem bromodomain displays a highly positive surface potential, favoring DNA interaction.
**(C)**Structural superimposition of the TAF1 tandem bromodomain (PDB: 1EQF) and the TAF1L tandem bromodomain (AlphaFold model: AF-Q8IZX4-F1), highlighting conservation of the basic patch in BD1. The RKR motif in TAF1 is replaced by an RKC motif in TAF1L, with a cysteine substituting for one of the arginines.


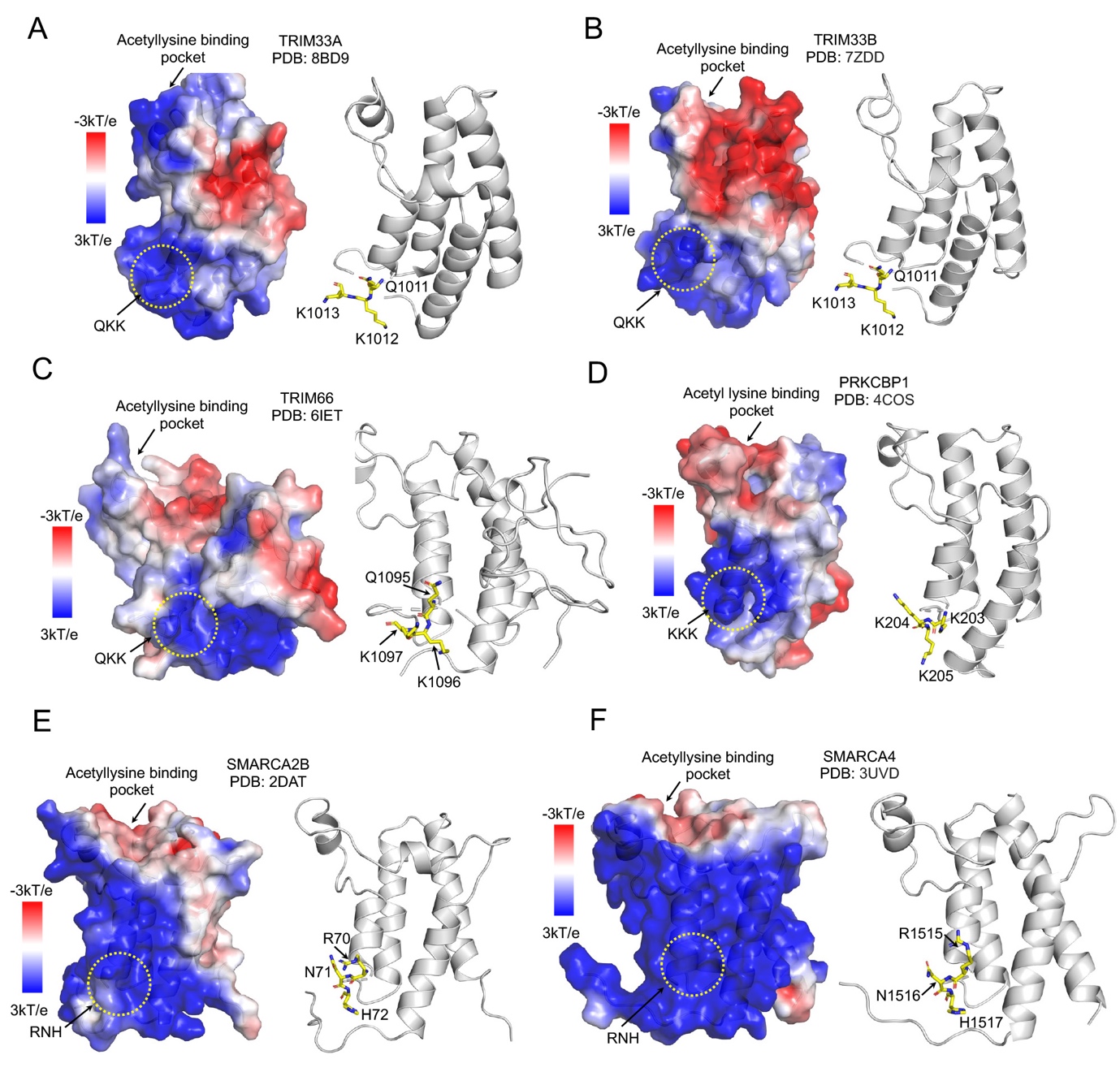


**Figure S8.** (A-F) Electrostatic surface potentials of TRIM33A (8BD9), TRIM33B (7ZDD), TRIM66 (6IET), PRKCBP1(4COS), SMARCA2B (2DAT), and SMARCA4 (3UVD). Basic patch residues are indicated with yellow circles, and the corresponding amino acid residues are shown in stick representation.


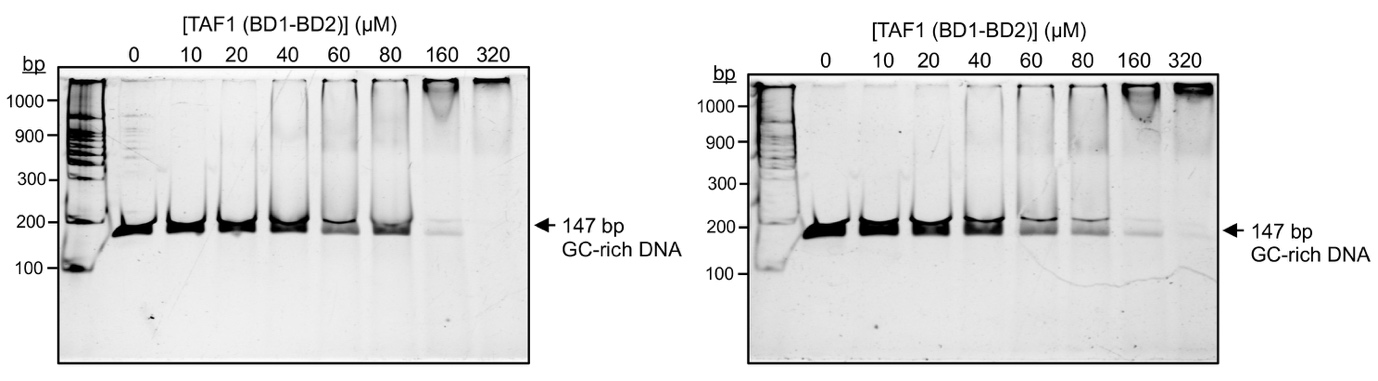


**Figure S9.** EMSA titrations of TAF1 tandem bromodomain with 147 bp GC-rich 601 DNA. Data from biological replicates are shown.


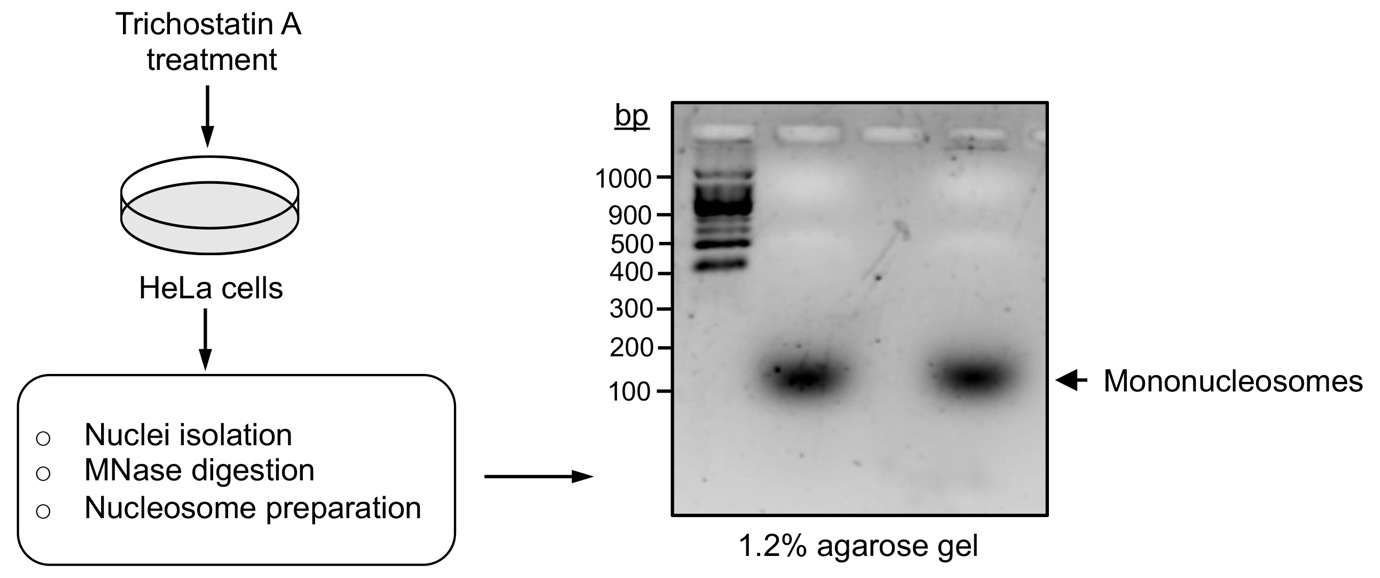


**Figure S10.** Mononucleosomes were isolated from HeLa cells and their integrity was assessed by electrophoresis on a 1.2% agarose gel.


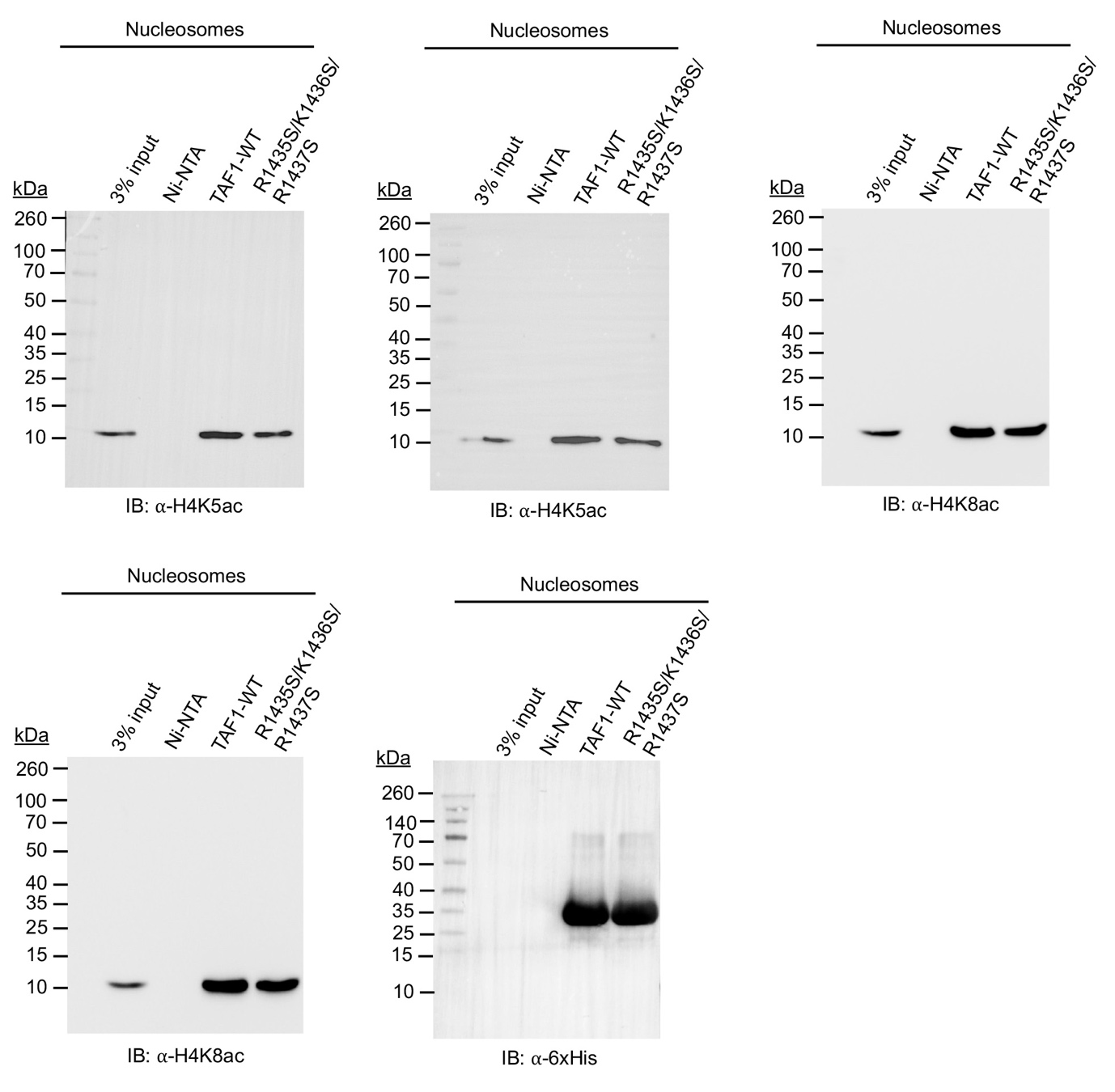


**Figure S11.** The BD1 mutant (R1435S/K1436S/R1437S) of the TAF1 tandem bromodomain exhibited a marked reduction in binding to acetylated mononucleosomes compared to wild-type TAF1, as detected using anti-H4K5ac and anti-H4K8ac antibodies. Immunoblotting with an anti-6xHis antibody confirmed equal loading of TAF1 proteins across the samples. Data shown are from biological duplicates (full uncropped western blots).

**Table S1.** List of the forward primers designed for site-directed mutagenesis. Reverse primers used are the reverse-complement to the given forward primers.


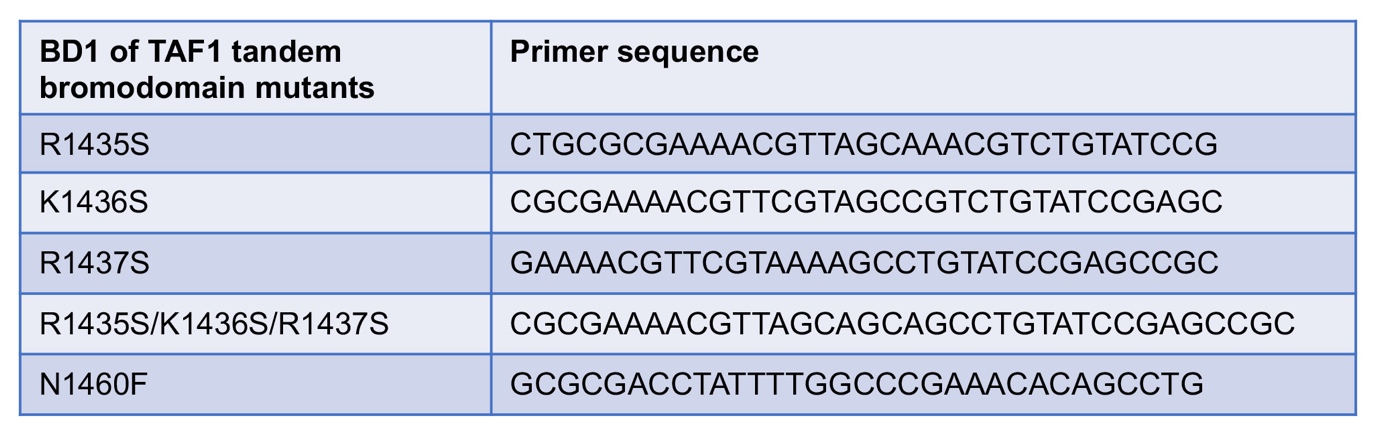

Supplement: Online supplementary material 1 [file bcj-482-19-BCJ20253064-s001.docx]
